# Asymptomatic Liver Abscesses Mimicking Metastases in Patients after Whipple Surgery: Infectious Complications following Percutaneous Biopsy—A Report of Two Cases

**DOI:** 10.1155/2012/817314

**Published:** 2012-09-03

**Authors:** Kan K. Zhang, Majid Mayody, Rajesh P. Shah, Efsevia Vakiani, George I. Getrajdman, Lynn A. Brody, Stephen B. Solomon

**Affiliations:** ^1^Division of Interventional Radiology, Department of Radiology, Memorial Sloan-Kettering Cancer Center (MSKCC), 1275 York Avenue, M276C, New York, NY 10065, USA; ^2^Stanford Hospital and Clinics, Stanford, CA 94305, USA

## Abstract

We present two cases of hepatic abscesses that mimicked metastases in patients having undergone Whipple surgery. Both patients had similar imaging features on computed tomographic (CT) scan and ultrasound, and at the time of referral for biopsy neither patient was clinically suspected to have liver abscess. Both patients underwent biopsy of liver lesions and developed postprocedural infectious complications.

## 1. Introduction

Cancer patients require frequent cross-sectional imaging to assess for progression of disease. The liver is a common site of metastasis for many cancers. New liver lesions in cancer patients are likely to be metastases and biopsy is commonly performed to confirm the diagnosis.

Less frequently, new liver lesions in cancer patients can have an infectious etiology. In patients with biliary, duodenal, or pancreatic cancer, Whipple surgery (pancreaticoduodenectomy) and other biliary interventions which remove or disrupt the sphincter of Oddi and allow bacterial colonization of the biliary tree increase the risk of hepatic abscess formation [1]. Liver-directed therapies can exacerbate infection in these high-risk patients [2]. 

Liver abscess typically is the result of a pyogenic or amoebic infection and generally cause symptoms including fever and leukocytosis. It is possible, however, that the episode of infection that preceded liver abscess formation either remains subclinical or coincides with other postoperative issues. So when new liver lesions are found on surveillance cross-sectional imaging studies, infection is rarely considered, particularly in the absence of suggestive clinical history and laboratory abnormalities. We present two cases of hepatic abscesses that mimicked metastases in patients having undergone Whipple surgery. Both patients had similar imaging features on computed tomographic (CT) scan and ultrasound, and at the time of referral for biopsy neither patient was clinically suspected to have liver abscess. Both patients underwent biopsy of liver lesions and developed postprocedural infectious complications.

## 2. Case Report 

### 2.1. Case 1

**Figure 1:**
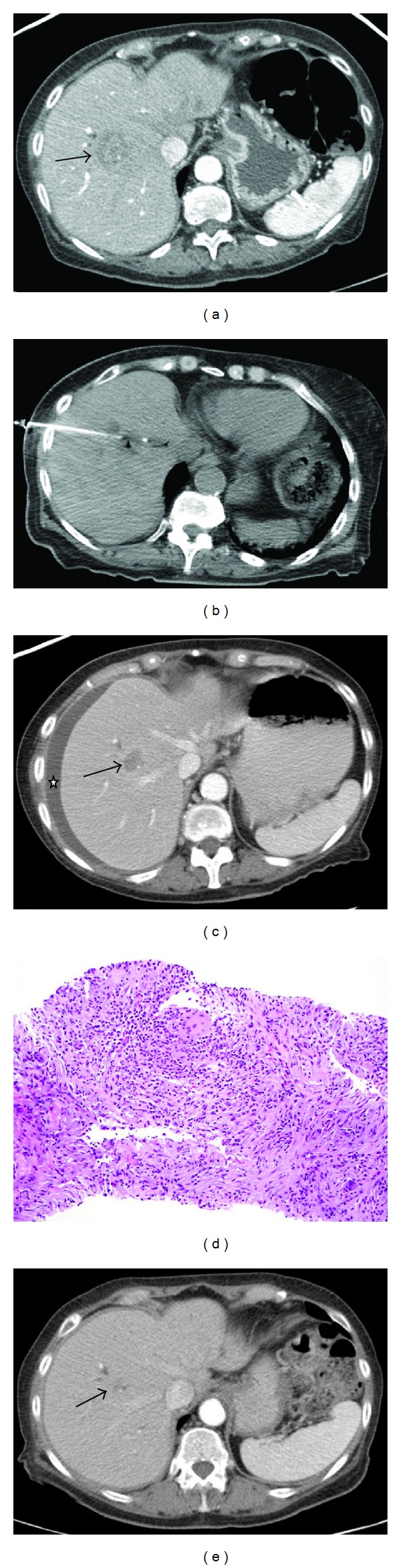
(a) Patient with history of cholangiocarcinoma status after pancreaticoduodenectomy. Axial contrast-enhanced CT image obtained a few weeks after surgery demonstrates one of the two new hepatic lesions in segment 8 (arrow). (b) Axial image from CT guided needle biopsy of the same lesion. The biopsy needle is placed within the lesion to obtain one of core samples. (c) Axial contrast-enhanced CT image one day after biopsy demonstrates new right perihepatic/subdiaphragmatic collection (asterix), which cultured positive for *E. coli*. The biopsied lesion is visible (arrow). (d) Histologic examination of core specimens shows a granuloma along with a mixed inflammatory infiltrate composed of lymphocyte, eosinophils, and neutrophils. (e) Contrast-enhanced CT image three months after biopsy shows resolution of liver lesions. The area of the biopsied segment 8 lesion is marked by an arrow.

A 73-year-old woman with history of Crohn's disease and cholangiocarcinoma invading the duodenum and pancreas underwent pancreaticoduodenectomy (PD). A surveillance CT scan performed several weeks after the surgery revealed two new hepatic lesions (Figure 1(a)). She had no clinical symptoms. She was referred to the interventional radiology service for biopsy of a liver lesion and for placement of a venous infusion port. Complete blood count, liver function tests, coagulation profile, and basic metabolic profile were all within normal limits. The liver biopsy was performed under moderate sedation with the patient in the supine position. Under CT guidance, a 2.5 cm lesion in segment 8 was accessed with a 19-gauge/20-gauge automatic core biopsy gun (Temno, CareFusion, Waukegan, IL) via a lateral intercostal approach (Figure 1(b)). A good core of tissue was obtained. A touch preparation was made by placing the core of tissue on a glass slide and rolling the specimen gently around the slide. The sample was immediately evaluated for adequacy by an on-site cytotechnologist. No neoplastic cells were seen on the initial touch preparation. Ultimately five core samples were obtained from different areas of the lesion; each appeared visually adequate, but none of the touch preparation samples contained neoplastic cells on the on-site evaluation. The biopsy was terminated based on CT imaging confirmation of adequate sampling of the lesion. The specimens were submitted for both cytopathologic and surgical pathologic evaluations, which revealed liver parenchyma with chronic active inflammation, granulomas, and a reactive bile ductule proliferation (Figure 1(d)). No carcinoma was seen. Special stains for mycobacteria and fungal organisms were negative. No microbiology specimens were sent due to lack of clinical suspicion for infectious etiology.

The patient developed right upper quadrant pain in the recovery area. She did not have any clinical signs of hemorrhage or sepsis. The pain was controlled with 50 micrograms of intravenous fentanyl. She remained hemodynamically stable throughout a three-hour postbiopsy observation period. The pain was attributed to minimal blood or bile leaking from the puncture site irritating the diaphragm. She was sent home on oral pain medications. The day after the biopsy, the patient continued to complain of right upper quadrant pain. She also developed fever and was brought back to the hospital for further evaluation. She had an elevated white blood cell count of 13.6 K/mcL, increased from prebiopsy value of 5.1 K/mcL. CT scan showed a new right perihepatic/subcapsular fluid collection. This was aspirated under CT guidance and yielded 600 mL of serosanguineous fluid (Figure 1(c)). The patient was discharged home with instructions regarding signs of hemorrhage and infection. No drainage catheter was placed due to the benign gross character of the fluid. Culture results returned positive for *Escherichia coli*. The patient was placed on oral ciprofloxacin. In the following 2 weeks, the patient's pain persisted and she developed dyspnea; a CT scan of chest was performed to rule out pulmonary embolism. This showed subsegmental bilateral pulmonary artery embolism and also enlarging, recurrent right perihepatic/subdiaphragmatic collection with new loculations. It was drained with an all-purpose drainage catheter and intracavitary alteplase instillation. Culture showed *Escherichia coli*. Pulmonary embolism was treated with anticoagulation therapy. The patient was treated and discharged with ciprofloxacin for her perihepatic infection. The collection resolved and the catheter was removed three weeks after placement. CT scan of the abdomen was repeated 3 months after the biopsy, which showed resolution of the hepatic lesions, including the lesion that was biopsied (Figure 1(e)). The patient had not received any chemotherapy since biopsy.

### 2.2. Case 2

**Figure 2:**
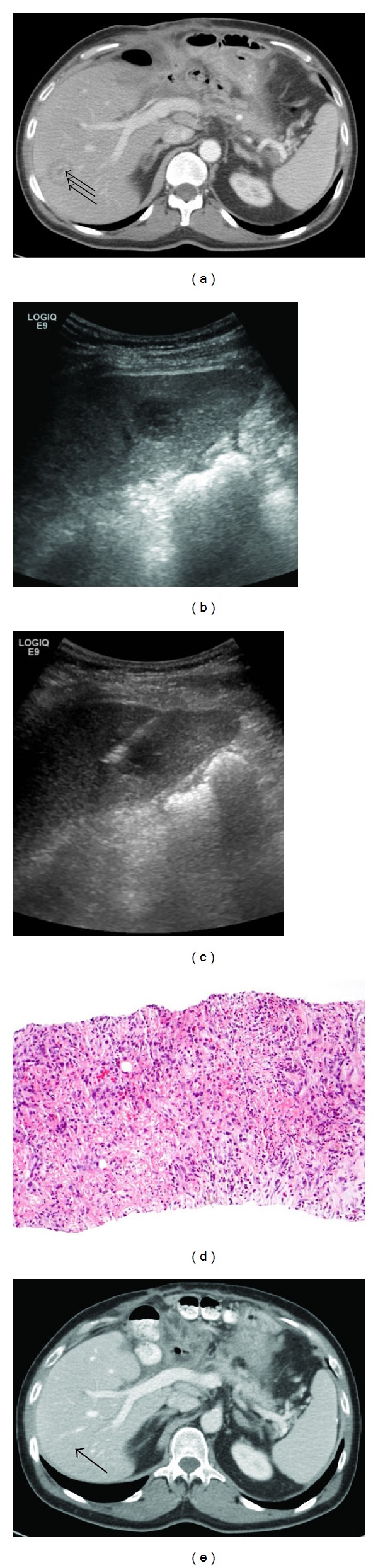
(a) Patient with history of pancreatic adenocarcinoma status after pancreaticoduodenectomy. Axial contrast-enhanced CT image obtained a few weeks after surgery showed three new liver lesions. This lesion in segment 8 has “double target sign” with three discrete layers (arrows). (b) Oblique image from ultrasound guided needle biopsy demonstrates another “double target sign” lesion in segment 6. (c) Biopsy needle within the segment 6 lesion. (d) Histologic examination of core specimens shows abundant neutrophils admixed with reactive fibroblasts. (e) Contrast-enhanced CT image three months after biopsy shows resolution of liver lesions. The area of the segment 8 lesion shown on (a) is marked by an arrow.

A 61-year-old man 2 months after Whipple surgery for pancreatic head adenocarcinoma developed three new hepatic lesions on CT scan (Figure 2(a)). He had an episode of postoperative abdominal infection which had resolved by the time of surveillance CT scan. He was referred to the interventional radiology service for biopsy of a liver lesion to confirm metastatic disease. Complete blood count, coagulation profile, and basic metabolic profile were all within normal limits. Liver function tests were unremarkable except for mild elevation of alkaline phosphatase to 176 Units/L (normal range 45–129 Units/L) and ALT to 56 Units/L (normal range 5–37 Units/L). 

Core biopsy was performed under ultrasound guidance with conscious sedation. Gray scale ultrasound images revealed an approximately 2.5 cm segment 6 liver lesion with “double target sign” appearance, similar to CT findings (Figure 2(b)). A 19-gauge/20-gauge automatic core biopsy gun (Temno, CareFusion,Waukegan, IL) was used to ultimately obtain 4 samples via a lateral intercostal approach (Figures 2(c) and 2(d)). None of the touch preparation slides of the core specimens revealed neoplastic cell on the on-site microscopic examination. The biopsy was terminated based on ultrasound imaging confirmation of adequate sampling of the lesion. Specimens were submitted for cytopathologic, microbiologic, and surgical pathologic evaluations which revealed a benign fibroinflammatory infiltrate consistent with organizing abscess and positive culture for *Escherichia coli* (Figure 2(d)). 

The patient developed rigors in the recovery area after biopsy and was admitted for intravenous antibiotic treatment. Blood cultures were negative and blood chemistries remained within normal limits during his hospital stay. The patient was discharged after five days to complete a 4-week course of intravenous ceftriaxone treatment. All liver lesions near-completely resolved on follow up CT scan three months after biopsy (Figure 2(e)). The patient did not receive any chemotherapy since biopsy.

## 3. Discussion

Liver abscess does not have any pathognomonic imaging appearance. Infectious etiology is generally suspected based on a combination of clinical symptoms, laboratory abnormalities, medical history, and corresponding imaging findings. 

Liver abscesses may appear as a unilocular central hypodense area with a hypovascular or hypervascular rim [3, 4]. A common appearance is a multiloculated cystic cavity with thin or thick enhancing walls [5]. They may contain gas or air-fluid levels [6]. A “cluster” sign is when small abscesses appear to cluster or aggregate together suggesting coalescence into a single large cavity [6]. A “double target” sign is caused by addition of perilesional edema or parenchymal hyperemia where there are at least three discernible layers including the central cavity [3, 4]. On ultrasound, a similar pattern is sometimes referred to as a “bull's eye” lesion [3, 7]. The double target sign is not specific to abscess. It is seen in 50% of abscesses but can be seen in up to 30% of hepatic malignancies [8–10]. 

However, when the infection and inflammation is more chronic, a central pus filled cavity may not form in such a lesion. Granulation tissues may organize into layers instead. Histologically, authors have described these lesions with central necrotic components containing polymorphonuclear leukocytes, surrounded by layers of granulation tissue [6] and possible edema in the outer periphery. This explanation seems appropriate for both of our patients where chronic ascending biliary infection is more likely due to removal of the Sphincter of Oddi during pancreaticoduodenectomy (PD). The liver lesions in both patients were proven to be infectious by lack of neoplastic histology, positive cultures, and improvement with antibiotic therapy. 

Liver abscesses are almost always symptomatic and are almost always associated with laboratory abnormalities [6, 11]. Among the most common presentations of liver abscesses are fever, chills, right upper quadrant pain, jaundice, leukocytosis, and abnormal liver functions tests. Asymptomatic liver abscess in a nonimmune compromised patient is extremely rare. A patient with asymptomatic pyogenic liver abscess had abnormal liver function and ultimately succumbed to fulminating sepsis [12]. Four patients with liver abscesses and pylephlebitis who were thought to have hepatocellular carcinoma were all febrile and had abnormal liver function [13].

Liver abscesses are often a result of amoebic, or bacterial infections [7, 11, 14]. *Escherichia coli*, likely from gastrointestinal origin, was the organism responsible for the infection in both of our patients. 

The mortality of PD has decreased drastically in the past decade, but morbidity remains steady [15]. One of the most serious and common complications is formation of liver abscess [1]. Post-PD patients are more prone to liver parenchymal infection due to removal of Sphincter of Oddi, which normally prevents gastrointestinal tract bacteria from entering the sterile biliary tree. After PD, the biliary tract is colonized with gastrointestinal flora, which can more easily cause liver abscess formation, especially after liver-directed therapies [1, 2]. Therefore, post-PD patients who have a high risk for liver metastases due to the nature and location of their primary tumors are also at high risk for hepatic abscess formation. 

Biopsy of infectious lesions in our patients likely caused dissemination manifested by infected perihepatic fluid collection in one and rigors in the other. The biopsy needle likely contracted bacteria from the abscess and seeded the organism in the surrounding parenchyma and blood stream during needle manipulation and withdrawal. While infections caused by more invasive liver directed therapies have been widely reported [2], those caused by biopsy have not been reported. 

Proper awareness on the part of the referring physician and the interventional radiologist about the possibility of an infectious etiology for new liver lesions helps setting an appropriate endpoint for a biopsy procedure. It also facilitates addressing of the potential postbiopsy infectious complications more effectively. 

Our cases may serve as precautionary examples that asymptomatic liver abscesses can mimic metastases, especially in patients after Whipple surgery who are more prone to both liver metastases and liver infections. Biopsy of these lesions may cause spread of infection. It may be wise to monitor these patients more closely for signs of postprocedural sepsis and infectious complications. Prophylactic antibiotics prior to biopsy should also be considered.
