# Corrigendum to “Asymptomatic Liver Abscesses Mimicking Metastases in Patients after Whipple Surgery: Infectious Complications following Percutaneous Biopsy—A Report of Two Cases”

**DOI:** 10.1155/2015/783506

**Published:** 2015-02-03

**Authors:** Kan K. Zhang, Majid Maybody, Rajesh P. Shah, Efsevia Vakiani, George I. Getrajdman, Lynn A. Brody, Stephen B. Solomon

**Affiliations:** ^1^Division of Interventional Radiology, Department of Radiology, Memorial Sloan-Kettering Cancer Center (MSKCC), 1275 York Avenue, M276C, New York, NY 10065, USA; ^2^Stanford Hospital and Clinics, Stanford, CA 94305, USA

In the paper titled “Asymptomatic Liver Abscesses Mimicking Metastases in Patients after Whipple Surgery: Infectious Complications following Percutaneous Biopsy—A Report of Two Cases” the misspelled last name of the 2nd author was “Mayody” and is corrected as above.

